# The efficacy of metaphylaxis in treatment of recurrent urolithiasis

**Published:** 2017

**Authors:** P Banov, E Ceban

**Affiliations:** * Department of Urology and Surgical Nephrology, “Nicolae Testimiţanu” State University of Medicine and Pharmacy, Chisinau, Republic of Moldova

**Keywords:** urolithiasis, metaphylaxis, recurrence prevention, specialized or general metaphylaxis treatment

## Abstract

Urolithiasis is a significant social and financial problem. According to contemporary literature data, 1-5% of the global population suffers from urolithiasis. The prevalence of this disease is about 10% of the population of the Republic of Moldova. Scientific and practical researches in the field of urology, and especially those devoted to renal lithiasis, focus on the diagnosis and treatment process, giving little importance to the cause of formation, metabolic disturbances, and especially to prophylaxis and metaphylaxis of the disease recurrence. However, the impact of this disease may be diminished by specialized or general metaphylaxis treatment. The article presents results of the analysis of different methods of metaphylaxis of recurrent urolithiasis. The implementation of metaphylaxis measures significantly reduces the rate and risk of recurrence in patients with recurrent urolithiasis. Specialized metaphylaxis treatment reduces the risk of lithiasis recurrence 5 times and general metaphylaxis - 2 times compared to the lack of metaphylaxis, which requires this treatment to prevent the recurrence of urolithiasis.

## Introduction

Urolithiasis currently occupies one of the leading places in the structure of urological diseases being qualified as a major medical-demographic problem [**1**-**5**]. Over the last decades, a progressive increase in the incidence of urolithiasis was recorded on the entire earth globe [**7**-**9**,**11**-**18**]. According to contemporary literature data, 1-5% of the global population suffers from urolithiasis, but the incidence of this pathology varies depending on geographical area. The risk of urolithiasis in adults is higher in the western hemisphere (5-9% in Europe, 12% in Canada, 13-15% in the USA) compared to the eastern hemisphere (1-5%), although the maximum incidence was registered in some Asian countries such as Saudi Arabia (20.1%) [**7**-**9**,**11**-**18**]. The prevalence of this disease is about 10% of the population from the Republic of Moldova [**6**,**10**]. The incidence of urolithiasis also depends on both racial distribution and socio-economic status of the surveyed population.

An annual increase of urolithiasis incidence in economically well-developed countries is 2-2.5% (Germany, USA, etc.), but in the Russian Federation, this index ranges from 1% to 3% [**11**-**15**,**18**]. There is also an increase of urolithiasis incidence and prevalence in the Republic of Moldova, which from 2005 has become the first in the structure of diseases in urological clinics, which places secondly some pathology as inflammations and prostate adenoma [**10**]. In male urolithiasis, males are affected more often, at a rate of 52-60%. Male/ female ratio is 1:3 or 1:2. Some authors, however, found much higher figures due to excessive working regimen, food abuse, urethro-prostatic disease and other factors that determine an increased prevalence of lithiasis in men [**11**-**15**,**18**].

In 1980s and 90s of the last century, the basic methods of treatment of urolithiasis were conservative methods and surgical techniques. At present, significant progress is, along with extracorporeal shock wave lithotripsy (ESWL), the implementation of modern endourological techniques [**1**-**3**] in clinical practice. These methods occupy one of the first places in the curative process.

Until now, great experience has been gained in the treatment of urolithiasis. The success of treatment is largely determined by the chosen tactics and curative method. The methods applied and their combination in multimodal treatment of pathology, result in total stone removal (stone-free) [**3**,**9**,**12**]. However, a detailed review of literature demonstrates that scientific-practical research in urology, and especially those devoted to renal lithiasis, focuses on the diagnosis and treatment process, giving little importance to the cause of formation, metabolic disturbances and in particular to prophylaxis and metaphylaxis of the disease recurrences [**4**,**5**]. After a thorough analysis of specialty literature on prophylaxis and metaphylaxis of urolithiasis recently published in PubMed electronic database, there has been an increase in the number of articles published in this field over the last 7 years [**1**,**2**].

In their study, Meneses JA et al. (2012) showed that in patients with recurrent urolithiasis pathology, who did not perform metaphylaxis measurements, both the occurrence of chronic renal disease and the recurrence rate were higher compared to patients who underwent metaphylaxis. The recurrence of disease leads to partial or total loss of renal functional capacity, which sometimes progresses to chronic renal failure and invalidity of patients with decreased ability to work and quality of life [**16**].

The diversity of the causes of calculi occurrence but also of clinical forms of urolithiasis, chemical composition, localization, present urinary infection, complicate the prophylaxis and metaphylaxis of this pathology, which needs to be viewed individually in each case as far as possible [**11**-**14**,**16**-**18**]. Until now, there has been no single opinion on the type and volume of metaphylaxis medical manipulation that would be optimal to be performed after the removal of urinary concretions.

The purpose of the research was to evaluate the efficacy of metaphylaxis in patients with recurrent urolithiasis. 

## Material and methods

The trial was conducted in the clinic of Urology and Surgical Nephrology, “Nicolae Testemitanu” State University of Medicine and Pharmacy, Republican Clinical Hospital during the years 2010 and 2014 based on clinical and laboratory data from 160 patients with recurrent kidney lithiasis, who were treated in inpatient and outpatient departments.

The inclusion criteria were the following: patients with recurrent renal lithiasis who signed the study participation consent. The exclusion criteria were the following: patients who refused to sign the study participation agreement or were diagnosed with Chronic Renal Disease K-DOQI degree IV-V and/ or other concomitant serious illnesses.

The study was approved by the Research Ethics Committee of “Nicolae Testemitanu” SUMPh, Chisinau, Republic of Moldova.

Patients were randomly assigned to research groups to determine the efficacy of metaphylaxis. Group I included 58 patients of total number of investigated patients who underwent a complex metabolic evaluation, after which, patients were prescribed individual metaphylaxis treatment recommendations. Group II included 52 patients with recurrent calculi, who were recommended after inpatient treatment and general measures against recurrence of urolithiasis. Group III included 50 patients with recurrent urolithiasis, who were treated in the Urology SCR Department, were questioned 3 years after the treatment, but failed to fulfill the disease prevention recommendations.

**Fig. 1:**
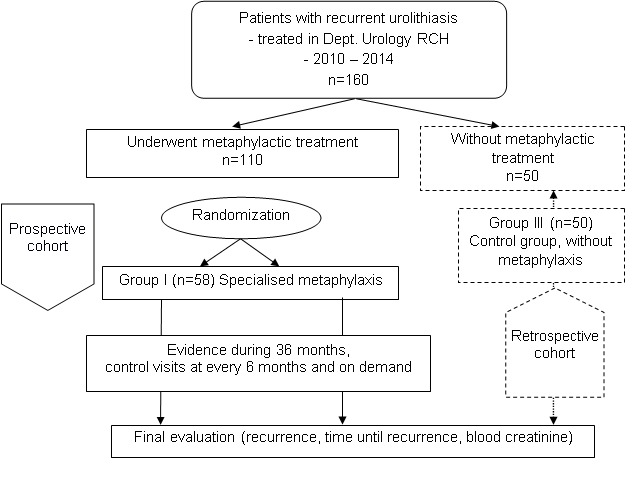
General design of study (CONSORT-chart)

The study design (CONSORT-chart) is shown in **Fig. 1**.

The comparative efficacy of the treatments was assessed by the following tests: Pearson’s chi-square, Hazard, Kaplan-Meier, RR - relative risk and NNT (Number Needed to Treat) - the number of patients necessary to treat to avoid an event. The data were processed by using statistical software package SPSS 21.

## Results and discussions

All 160 (100%) patients completed the study. According to age distribution, 10.6% of the cases were between 18 and 30 years old, 65.6% - 31 and 60 years old and 23.8% - over 60 years old (**Table 1**).

**Table 1:** Distribution of patients by gender and age

			Number of patients			
Patients’ age	Males		Females		Total	
	n	%	n	%	n	%
18-30 years old	6	3,8%	11	6,9%	17	10,6%
31-60 years old	38	23,7%	67	41,8%	105	65,6%
Over 60 years old	10	6,3%	28	17,5%	38	23,8%
Total	54	33,8%	106	66,2%	160	100%

Analyzing **Table 1**, we noticed that renal lithiasis affects predominantly between 30 and 60 years old, accounting for 65.6% (105 out of 160) of the cases, which confirmed the social impact of the pathology affecting the working life period.

The distribution of patients on research groups is presented in **Table 2**. 

**Table 2:** Patients’ distribution in research groups

Parameters		Group I	Group II	Group III	Total	Pearson’s χ2 (DF)	p
Gender	Males	19	14	21	54	2,631 (2)	0,268
	Females	39	38	29	106		
Total		58	52	50	160		
Age (years)	18-30	6	6	5	17	0,259 (4)	0,992
	31-60	39	35	31	105		
	Over 60	13	11	14	38		
Total		58	52	50	160		
Treatment applied	ESWL	21	21	20	62	0,259 (4)	0,992
	PLT	25	21	20	66		
	URS	12	10	10	32		
Total		58	52	50	160		
*Note:* ESWL = extracorporeal shock waves lithotripsy, PLT = Pyelolithotomy, URS = ureteroscopy, DF = Degree of Freedom.							

Analyzing the data in **Table 2** we noted that the distribution of patients by groups, according to age and method of treatment previously applied, which was homogeneous, the groups were comparable and the statistical significant differences between study groups were not found (p > 0.05).

During the follow-up (36 months) of patients with recurrent urolithiasis from those three groups (160 patients) included in the study, recurrence of urolithiasis was detected in 42 patients (from the whole cohort) (**Table 3**).

**Table 3:** Rate of recurrence of urolithiasis in investigated group

Group	Recurrent	Non recurrent	Total	χ2	p
	n (%)	n (%)			
Group I	7 (12,1%)	51 (87,9%)	58	14,2	0,0008
Group II	13 (25,0%)	39 (75,0%)	52		
Group III	22 (44,0%)	28 (56,0%)	50		
Total	42 (26,3%)	118 (73,7%)	160		

Distribution of urolithiasis recurrence rates between the investigated groups was significantly non-homogeneous (χ2 (2) = 14.2, p = 0.0008).

**Fig. 2:**
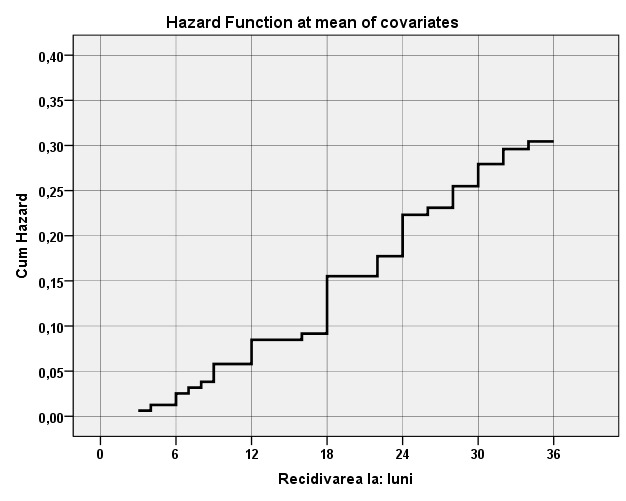
Hazard rate function of urolithiasis recurrence appearance for all patients included in the trial (months)

Hazard rate (HR) function of urolithiasis recurrence appearance for the three studied groups is shown in **Fig. 2**.

As shown in **Fig. 2**, the risk of recurrence increased concurrently with the time interval increase from the removal of recurrent calculus. Therefore, the risk of recurrence appearance in patients with recurrent urolithiasis one year after the calculus removal was of about 8%, in two years – of about 22%, and in three years of about 30%. 

The lowest rate of urolithiasis recurrence occurred in Group I patients, accounting for 12.1% (7 out of 58 patients). In 13 patients (25.0%) in Group II (n = 52) the recurrence of urolithiasis was determined. The highest rate of recurrence occurred in Group III patients and accounted for 44.0% (22 out of 50 patients).

The relative risk of urolithiasis recurrence in patients who, after the metabolic analysis, followed the specialized metaphylaxis recommendations (Group I) was RR=0.22 (95%CI=0.09-0.51; χ2(2)=13.9; p=0.0001). The data obtained (**Table 4**) showed that the risk of urolithiasis recurrence in Group I patients was 4.55 times lower compared to Group III, with a statistical significance (p = 0.0001).

**Table 4:** Relative risk of urolithiasis recurrence in studied groups

Group	RR	CI 95%	χ2	p
Group I (n=58)	0,22	0,09 – 0,51	13,9	0,0001
Group II (n=52)	0,495	0,24 – 0,98	4,08	0,044
Group III (n=50)	-	-	-	-
*Note:* RR = relative risk, CI = confidence interval, χ2 = Pearson’s chi-square, compared to Group III				

The relative risk of recurrence of urolithiasis in patients who met metaphylaxis general recommendations (Group II) was RR = 0.495 (CI 95% 0.24-0.98; x2 (2) = 4.08; p = 0.044). Based on the obtained results (**Table 4**), it was determined that the risk of recurrence of urolithiasis in this group of patients (Group II) was 2.02 times lower compared to the risk in Group III patients, with a statistical significance (p = 0.044). Although insignificant, the relative risk of recurrence development was higher in group II patients compared to patients who met specialized metaphylaxis recommendations, which outlined the anti-recurrent efficacy of these measures.

**Fig. 3:**
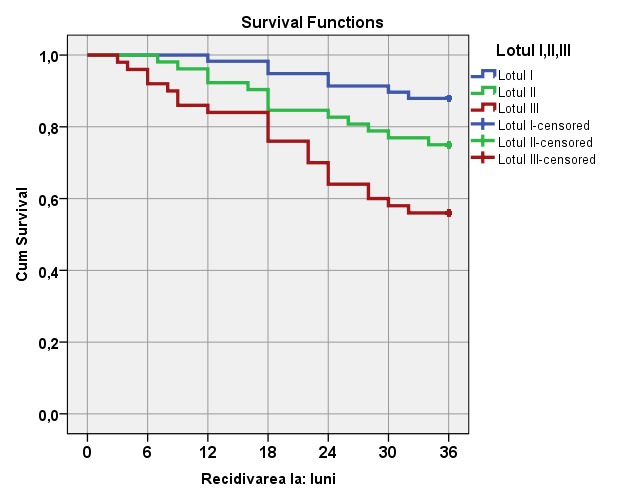
Kaplan-Meier curves of urolithiasis recurrence appearance in three investigated groups (months)

By using Kaplan-Meier curves, the time to recurrence of urinary calculi in the investigated and control groups was compared, determining the differences concerning the metaphylaxis treatment applied methods (**Fig. 3**).

The differences between “time to recurrence” in the investigated groups are statistically significant, as evidenced by log-rank test (**Table 5**).

**Table 5:** Average time until stone recurrence

Groups	Mean (months)	Standard Deviation	95% Confidence Interval		Log-rank test (Mantel-Cox)	P
			Upper limit	Lower limit		
Group I	34,379	0,648	33,110	35,649	15,067	0,0001
Group II	31,846	1,145	29,602	34,090		
Group III	27,860	1,538	24,845	30,875		
Total	31,519	0,685	30,176	32,862		
*Note:* Log-rank test (Mantel-Cox) for global comparison						

While comparing pairs between specialized metaphysics (group I) and those with general metaphylaxis (group II), it was determined that the differences in the recurrence-free interval of urolithiasis were insignificant and the meaningful tests performed between these groups revealed p value > 0,05 (**Table 6**).

**Table 6:** Results of pairs comparisons to recurrence rate between the investigated groups

Pairs comparisons		Group I		Group II		Group III	
		χ2	p	χ2	p	χ2	p
Log Rank test (Mantel-Cox)	Group I			3,261	0,071	14,965	0,000
	Group II	3,261	0,071			4,261	0,039
	Group III	14,965	0,000	4,261	0,039		

In order to express the likelihood of general and specialized metaphysical treatments benefit, the number of patients to be treated in the study, i.e. the necessary number of patients required to be treated (NNT) to prevent the recurrence of urolithiasis, was calculated as reciprocal of absolute risk reduction of developing a recurrence of urinary lithiasis.

Thus, when calculating the absolute risk of urolithiasis recurrence in the control group for 3 years, it was determined that it represented 44%. At the same time, the absolute risk value in the group of patients benefiting from specialized metaphylaxis treatment was 12.07%. Therefore, the absolute risk reduction, defined as the absolute difference between urolithiasis recurrence rate in patients who underwent a specialized metaphylaxis treatment and a control group event rate, was 31.93%, with a confidence interval (CI 95%) within the limit of 15.82% to 48.04%. When calculating the required treatment number, its value, equal to 4, was obtained, which meant that one of four patients benefitted from a specialized metaphylaxis treatment, fact expressed in the absence of the recurrence of urinary lithiasis, compared to patients in the control group. The confidence interval (CI 95%) for the required number to be treated was 2.1-6.3.

In the group of patients who underwent a general metaphylaxis treatment, 25% of them developed a recurrence of urolithiasis, this percentage indicating the absolute risk value. Thus, compared to the control group, general metaphylaxis treatment reduced the absolute risk of developing recurrences of urinary lithiasis by 19%, the confidence interval (CI 95%) for this index being from 0.89% to 37.7%. The NNT index for this type of treatment was 6, with a confidence interval (CI 95%) from 2.7 to 111.8. In order to prevent the development of recurrence of urolithiasis, it was necessary that 6 patients underwent a general metaphylaxis treatment.

## Conclusion

The recurrence rate of recurrent urolithiasis was frequent and increases with time more and more after the removal of renal calculus, and the risk of recurrence in patients with recurrent urolithiasis one year after the calculus removal was of around 8%, after two years – of about 22%, and after three years of approximately 30%. The implementation of metaphylaxis measures reduced the rate and risk of recurrent urolithiasis. Specialized metaphylaxis treatment reduced risk 5 times, and generally 2 times compared to the lack of metaphylaxis, which required the need for this treatment to prevent recurrences.
